# Authentic Inner Compass and Subjective Well‐Being Among Chinese Emerging Adults: A Moderated Mediation Model

**DOI:** 10.1002/pchj.70000

**Published:** 2025-04-24

**Authors:** Niting Guo, Tianyuan Li

**Affiliations:** ^1^ School of Humanities and Social Science The Chinese University of Hong Kong Shenzhen P. R. China

**Keywords:** anxious attachment, authentic inner compass, resilience, self‐determination theory, subjective well‐being

## Abstract

Originating from the self‐determination theory, the authentic inner compass (AIC) enlightens people about their authentic values, interests, and life aspirations, functioning as an action‐guiding schema. Previous studies have examined AIC among adolescents, but its significance for emerging adults is underexplored, especially in less autonomy‐oriented cultures (e.g., Chinese cultures). Informed by the self‐determination theory, the present study aimed to investigate the association between AIC and subjective well‐being among Chinese emerging adults and examine further the mediating role of resilience and the moderating role of anxious attachment. A total of 155 Chinese emerging adults completed measures on AIC, resilience, anxious attachment, and subjective well‐being. The results showed that AIC was positively associated with subjective well‐being, and resilience fully mediated the association. Moreover, anxious attachment strengthened the positive link between AIC and resilience. The findings highlight the importance of AIC and resilience in boosting subjective well‐being, emphasizing the significance of AIC for anxiously attached individuals. The moderated mediation model enriches the self‐determination theory and resilience literature. Future practices aiming to promote the well‐being of Chinese emerging adults may focus on fostering AIC and resilience and consider individual differences in attachment styles.

## 1 Introduction

Self‐determination theory (Ryan and Deci 2017) posits that autonomy is one of three basic psychological needs essential for optimal human functioning and well‐being. Within this framework, authentic inner compass (AIC)—defined as a set of self‐directed values, interests, and goals informing individuals of what is truly important to them and serving as an action‐guiding schema—emerges as a crucial construct facilitating autonomy satisfaction (Assor 2018). When individuals possess a well‐developed AIC, they are better equipped to make autonomous choices aligned with their authentic preferences, which facilitates the satisfaction of their autonomy needs (Assor et al. 2023) and further contributes to subjective well‐being (SWB; Ryan and Deci 2017). While research has increasingly explored the relationship between AIC and SWB (e.g., Assor et al. 2021; Russo‐Netzer and Shoshani 2020), these studies have predominantly focused on Western, autonomy‐oriented cultures and adolescent populations. The role of AIC in less autonomy‐oriented cultural contexts, such as China, and its impact on different developmental stages remain underexplored. The related mechanisms and boundary conditions are also understudied. Thus, the current study aimed to investigate the association between AIC and SWB among Chinese emerging adults. Moreover, the mediating role of resilience and the moderating effect of anxious attachment were also examined.

### 1.1 The Role of AIC in Chinese Emerging Adults

Emerging adulthood, spanning ages 18 to 29, is a distinct developmental phase marked by identity exploration, instability, self‐focus, and feelings of being in‐between (Arnett 2000). During this transitional period, individuals navigate critical life decisions amid significant changes in education, career, and relationships. This developmental period has been validated in the Chinese context, with studies demonstrating robust construct validity for emerging adulthood measures among Chinese populations (Kuang et al., 2023, 2024). Moreover, the traditional Confucian concept of “standing firm at thirty” (三十而立), which emphasizes realizing one's life purpose and reaching independence by age 30, reflects the culturally rooted developmental expectations placed on Chinese emerging adults.

It is particularly meaningful to investigate the role of AIC in the population of Chinese emerging adults. On the one hand, Chinese emerging adults face not only the typical developmental tasks of identity formation and critical life decision‐making but also the cultural pressure of “standing firm at thirty.” In this context, AIC serves as a crucial resource. By clarifying what truly matters, AIC fosters self‐congruent decision‐making and strengthens identity commitment (Assor et al. 2023), which can empower emerging adults to navigate their developmental challenges. On the other hand, the sociocultural environment in China presents obstacles to the development of AIC. The examination‐oriented education system, emphasizing academic achievement, often limits opportunities for young individuals to explore their authentic interests and values (Kirkpatrick and Zang 2011). Furthermore, the cultural emphasis on collective harmony and social conformity can constrain personal autonomy, making it difficult for individuals to identify and act on their authentic preferences (Tweed and Lehman 2002; Hofstede 2011). The dual pressures of developmental tasks and sociocultural expectations call for further investigation of the role of AIC in Chinese emerging adults.

### 1.2 The Association Between AIC and SWB

Self‐determination theory provides a theoretical framework for understanding how AIC contributes to well‐being through autonomy satisfaction. Autonomy is a fundamental psychological need “to self‐organize and regulate one's own behavior, which includes the tendency to work toward inner coherence and integration among regulatory demands and goals” (Deci and Ryan 2000, p. 252). Self‐determination theory also suggests that a key way to achieve self‐regulation is to develop intrinsic, self‐aligned goals and aspirations (Ryan and Deci 2017). According to the self‐determination theory, autonomy satisfaction promotes psychological growth, optimal functioning, and well‐being (Ryan and Deci 2017). As an internal guide, AIC facilitates autonomy satisfaction by helping individuals identify and pursue values, interests, and goals that resonate with their authentic selves (Assor 2018; Assor et al. 2023), which further enhances SWB through increased self‐integration and psychological growth (Ryan and Deci 2017).

Extensive empirical research supports the positive relationship between AIC and SWB, particularly in autonomy‐oriented cultures. For example, Russo‐Netzer and Shoshani (2020) found that AIC was positively associated with SWB, with prioritization of meaning and positivity mediating the relationship. In a series of studies, AIC has been shown to predict increased subjective vitality, self‐esteem, and reduced depressive symptoms (Assor et al. 2021). AIC has also been linked to resistance to negative pressure (Assor et al. 2020a), highlighting its protective role in maintaining psychological functioning. Emerging evidence suggests that the benefits of AIC extend beyond autonomy‐oriented cultures. For instance, Cohen and Slobodin (2022) found that among Bedouin teaching students from authoritarian, hierarchical, and group‐oriented family structures, AIC, high need satisfaction, and low need frustration were linked to lower distress and greater positive affect. Similarly, S. Yu et al. (2024) found that AIC positively predicted psychological growth and well‐being among Chinese college students, suggesting that the adaptive function of AIC extends to collectivist cultural contexts (Hofstede 2011). Building on the theoretical and empirical foundation, we hypothesized that AIC would be positively associated with SWB among Chinese emerging adults.

### 1.3 The Mediating Role of Resilience

Resilience, defined as the ability and process of successfully adapting when faced with challenges (Masten 2018), might mediate the association between AIC and SWB. First, resilience can be bolstered by some psychological assets brought by AIC, such as positive emotions, self‐esteem, a sense of purpose, and effective coping skills (Mouatsou and Koutra 2021; Fergus and Zimmerman 2005; Rutten et al. 2013). Specifically, research has shown that AIC enhanced individuals' sense of meaning and self‐esteem (Assor et al. 2021; Russo‐Netzer and Shoshani 2020) and increased the resistance to negative pressure (Assor et al. 2020a). Furthermore, by enabling individuals to align their actions with authentic interests and values, AIC facilitates autonomy satisfaction, fostering positive emotion and resilience (Cohn et al. 2009; Vansteenkiste and Ryan 2013; Xu et al. 2021).

Meanwhile, there is a well‐established link between resilience and SWB. Resilient individuals tend to maintain higher levels of positive affect and life satisfaction as they possess effective coping strategies and the ability to bounce back from setbacks (Chen 2016; Satici 2016). By equipping individuals with the skills and resources to overcome adversities, resilience has been consistently shown to predict various aspects of well‐being (e.g., Hu et al. 2015; Smith et al. 2013). Moreover, resilience has also been reported to mediate the relationship between the need for self‐fulfillment and SWB (Yin et al. 2023). According to the self‐determination theory, decisions and actions that fulfill the individuals' fundamental needs generate positive emotions, which enhance well‐being by fostering resilience (Cohn et al. 2009; Vansteenkiste and Ryan 2013). Therefore, we postulated that resilience would mediate the association between AIC and SWB.

### 1.4 The Moderating Role of Anxious Attachment

We further proposed anxious attachment to moderate the AIC‐resilience relationship. Resilience is supported by both internal and external resources (Fergus and Zimmerman 2005; Masten 2018). Internal resources such as self‐esteem, sense of purpose, and stress resistance (Mouatsou and Koutra 2021; Fergus and Zimmerman 2005; Rutten et al. 2013) can be enhanced through AIC. External resources, including family connections and healthy interpersonal relationships (Feeney and Collins 2015; Windle 2011), are significantly influenced by attachment style (Bowlby 1982). Despite the recognized importance of both internal and external resources, few studies have investigated how these two types of resources interact to support resilience. We expected attachment style to interact with AIC to influence resilience.

Anxious attachment can significantly impair access to external resilience resources. According to the attachment theory (Bowlby 1982), early caregiving experiences shape individuals' internal working models that guide interpersonal relationships throughout life, thereby influencing access to external resilience resources. Anxious attachment is characterized by a pervasive fear of abandonment and a preoccupation with relationships, stemming from inconsistent caregiving (Mikulincer and Shaver 2019). This leads to excessive reassurance‐seeking, heightened emotional vulnerability, and emotional dysregulation in face of perceived relational threats, which undermine interpersonal relationships (Feeney and Fitzgerald 2019; Mikulincer and Shaver 2019). These interpersonal difficulties limit their access to social support and other external resources crucial for resilience (So and Fiori 2022). Empirical evidence also supports that anxious attachment is associated with lower resilience (Bender and Ingram 2018; Kural and Kovacs 2021).

In contrast, AIC primarily enhances internal resources for resilience, including self‐esteem, sense of meaning, and stress resistance (Assor et al. 2020a, 2021; Russo‐Netzer and Shoshani 2020). These internal resources become particularly crucial for individuals with anxious attachment who lack external support systems. When external resources are limited, the internal resources provided by AIC may play a vital compensatory role in building resilience. For individuals with low anxious attachment who can readily access external resources, the internal resources provided by AIC may be relatively less critical for resilience. Accordingly, we posited that anxious attachment would moderate the association between AIC and resilience.

### 1.5 The Present Study

To conclude, the present study sought to examine the extent to which AIC is correlated with SWB among Chinese emerging adults. Specifically, we investigated the mediating role of resilience in this relationship and explored whether anxious attachment moderates the association between AIC and resilience. This research aimed to clarify the mechanisms through which AIC influences SWB. We developed the following hypotheses:

Hypothesis 1 *AIC would be positively associated with SWB*.

Hypothesis 2 *Resilience would mediate the association between AIC and SWB*.

Hypothesis 3 *Anxious attachment would moderate the association between AIC and resilience such that the positive association would be more pronounced among individuals with a higher level of anxious attachment*.

**FIGURE 1:**
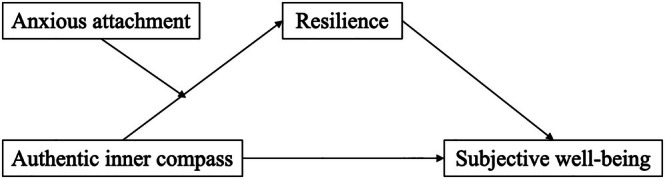
The moderated mediation model. Controlling for sex, age, educational level, and financial satisfaction. *p < 0.05, **p < 0.01, ***p < 0.001.

The conceptual model is presented in Figure 1.

## 2 Method

### 2.1 Participants and Procedure

This study employed convenience sampling to recruit participants through social media platforms such as WeChat. The data were collected via Wenjuanxing, an online survey platform, from April to May 2022. In line with the definition of emerging adulthood, the inclusion criteria focused on Chinese individuals aged 18 to 29. Responses were excluded if the questionnaire completion time was less than two minutes to ensure data quality. G‐Power 3.1 (Faul et al. 2007) was used to calculate an adequate sample size. Based on an *α* level of 0.05 and a power of 0.80, a sample size of 150 was needed to detect a small to medium effect size of 0.04 in regression analyses. The final sample comprised 155 Chinese emerging adults. All participants completed informed consent. The present study obtained ethical review approval from the City University of Hong Kong.

### 2.2 Measures

#### 2.2.1 Demographic Information

Participants provided information on sex (0 = *male*, 1 = *female*), age, educational level (1 = *below associate degree*, 2 = *associate degree*, 3 = *bachelor degree*, 4 = *master degree*, 5 = *doctorate*), and completed a question on financial satisfaction using a 7‐point Likert scale (1 = *strong dissatisfaction*; 7 = *strong satisfaction*). Considering regional differences in income levels, we did not collect the income information.

#### 2.2.2 AIC

The Chinese Version of the Authentic Inner Compass Scale was used to measure the level of AIC (Assor 2018; S. Yu et al. 2021). This scale contains eight items (e.g., “I have values that reflect the kind of person I truly want to be”) rated on a 7‐point Likert scale (1 = *almost never true*; 7 = *almost always true*). A higher total score indicates a higher level of AIC. This scale showed high reliability in Chinese samples (Assor et al. 2021; S. Yu et al. 2024). The Cronbach's alpha was 0.87 in the present study.

#### 2.2.3 SWB

The Satisfaction With Life Scale (Diener et al. 1985) was utilized to assess SWB with five items (e.g., “In most ways, my life is close to my ideal”). It was rated on a 7‐point Likert scale (1 = *strongly disagree*; 7 = *strongly agree*), with a higher score indicating higher SWB. This measure has been validated among the Chinese population (e.g., Chang et al. 2019). The Cronbach's alpha in this study was 0.90.

#### 2.2.4 Resilience

The 10‐item Connor–Davidson Resilience Scale (Campbell‐Sills and Stein 2007) was used to measure resilience. A sample item is “Can deal with whatever comes.” Participants responded on a 5‐point Likert scale (0 = *not true at all*; 4 = *true nearly all the time*), with higher scores indicating greater resilience. This 10‐item version has shown excellent reliability in Chinese samples (Cheng et al. 2020; N. X. Yu et al. 2021). The Cronbach's alpha in the current study was 0.89.

#### 2.2.5 Anxious Attachment

The 6‐item subscale assessing anxious attachment of the Revised Adult Attachment Scale (Collins 1996) was used. A sample item is “I often worry that other people don't really love me.” Participants rated the items on a 5‐Likert scale (1 = *not at all characteristic of me*; 5 = *very characteristic of me*), with a higher score indicating a greater anxious attachment style. This scale has shown good reliability (e.g., Li et al. 2020). The Cronbach's alpha was 0.90 in the present study.

### 2.3 Statistical Analysis

First, we calculated descriptive statistics and correlations of the key variables. Second, Model 4 of the PROCESS macro for SPSS v4.0 was applied to examine the mediating role of resilience in the association between AIC and SWB. Third, Model 7 of the PROCESS macro was applied to test the moderated mediation, setting anxious attachment as the moderator of the association between AIC and resilience. We controlled age, sex, educational level, and financial satisfaction in the mediation and moderated mediation analyses. The bootstrapping sample size was established at 5000, and the 95% confidence interval (95% CI) that did not encompass 0 suggested a statistically significant result. Employing the pick‐a‐point method, a simple slope analysis was conducted to illustrate the moderation effects of anxious attachment. We used SPSS version 26.0 to conduct the statistical analyses.

## 3 Results

### 3.1 Descriptive Statistics and Bivariate Correlations

Table 1 shows the demographic characteristics of the participants and the correlations among the variables. AIC was positively correlated with SWB (*r* = 0.24, *p =* 0.003), supporting Hypothesis 1. Besides, AIC was positively correlated with resilience (r = 0.46, *p* < 0.001) and negatively correlated with anxious attachment (*r* = −0.17, *p* = 0.037). Resilience showed a positive correlation with SWB (*r* = 0.47, *p* < 0.001) and a negative correlation with anxious attachment (*r* = −0.23, *p =* 0.004). There was also a negative correlation between anxious attachment and SWB (*r* = −0.26, *p* = 0.001).

**TABLE 1:** Descriptive statistics and bivariate correlations for variables (*N* = 155).

Variables	*Mean*	SD	1	2	3	4	5	6	7
1. Sex (0 = male, 1 = female)	—	—	—						
2. Age	24.25	3.94	−0.04	—					
3. Education level	2.99	0.63	0.08	0.13	—				
4. Financial satisfaction	3.94	1.34	−0.07	0.09	0.02	—			
5. Authentic inner compass	5.38	0.94	−0.02	0.08	0.20*	0.13	—		
6. Subjective well‐being	4.31	1.21	0.06	−0.07	0.08	0.48**	0.24**	—	
7. Resilience	3.67	0.59	−0.14	0.01	0.10	0.26**	0.46**	. 47**	—
8. Anxious attachment	2.52	0.96	−0.08	−0.26**	−0.11	−0.22**	−0.17*	−0.26**	−0.23**

*Note:* SD = standard deviation. *p < 0.05, **p < 0.01.

### 3.2 Mediation Model

In the mediation model (Table 2), the associations between AIC and resilience (*B* = 0.27, SE = 0.05, *p* < 0.001, 95% CI = [0.18, 0.36]) as well as the association between resilience and SWB (*B* = 0.75, SE = 0.16, *p* < 0.001, 95% CI = [0.44, 1.05]) were significantly positive after controlling for sex, age, educational level, and financial satisfaction. With resilience as a mediator, the indirect effect of AIC on SWB via resilience was significant (*B* = 0.20, SE = 0.07, 95% CI = [0.09, 0.35]), while the direct effect was non‐significant (*B* = 0.04, SE = 0.10, 95% CI = [−0.15, 0.23]), indicating that resilience fully mediated the association between AIC and SWB, supporting Hypothesis 2.

**TABLE 2:** Regression models testing the mediation effect of resilience on the association between authentic inner compass and subjective well‐being.

Outcome	Predictor	*R* ^2^	*F*	*B*	SE	95%CI
Subjective well‐being	Sex	0.29	12.27	0.20	0.17	[−0.14, 0.54]
	Age			−0.04	0.02	[−0.08, 0.00]
	Educational level			0.09	0.14	[−0.18, 0.36]
	Financial satisfaction			0.43***	0.06	[0.31, 0.56]
	Authentic inner compass			0.24*	0.09	[0.06, 0.42]
Resilience	Sex	0.27	11.11	−0.15	0.08	[−0.32, 0.02]
	Age			−0.01	0.01	[−0.03, 0.01]
	Educational level			0.03	0.07	[−0.10, 0.16]
	Financial satisfaction			0.09**	0.03	[0.03, 0.15]
	Authentic inner compass			0.27***	0.05	[0.18, 0.36]
Subjective well‐being	Sex	0.39	15.60	0.31	0.16	[−0.01, 0.63]
	Age			−0.03	0.02	[−0.07, 0.01]
	Educational level			0.07	0.13	[−0.18, 0.32]
	Financial satisfaction			0.36***	0.06	[0.24, 0.48]
	Authentic inner compass			0.04	0.10	[−0.15, 0.23]
	Resilience			0.75***	0.16	[0.44, 1.05]

*Note:* *p < 0.05, **p < 0.01, ***p < 0.001.

### 3.3 Moderated Mediation Model

**FIGURE 2:**
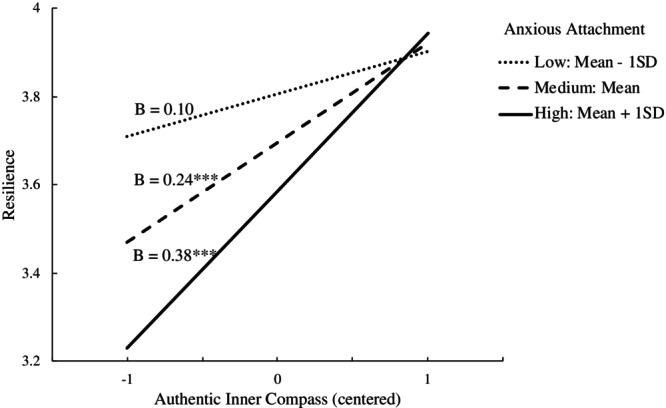
Moderation effect of different levels of anxious attachment in the association between authentic inner compass and resilience.

In the moderated mediation (Table 3), anxious attachment moderated the association between AIC and resilience (*B* = 0.15, SE = 0.05, *p* = 0.004, 95% CI = [0.05, 0.24]) after controlling for age, sex, educational level, and financial satisfaction, supporting Hypothesis 3. An extra 3.87% of the variance in resilience was explained by the interactions of AIC with anxious attachment. The association between AIC and resilience was non‐significant among individuals with low levels (mean – 1SD) of anxious attachment (*B* = 0.10, SE = 0.07, *p* = 0.134, 95% CI = [−0.03, 0.24]). However, it was significant among individuals with medium (*B* = 0.24, SE = 0.04, *p* < 0.001, 95% CI = [0.16, 0.33]) and high levels (mean + 1SD) of anxious attachment (*B* = 0.38, SE = 0.06, *p* < 0.001, 95% CI = [0.26, 0.50]). As illustrated in Figure 2, the moderation effects were more pronounced with higher levels of anxious attachment.

**TABLE 3:** Regression model testing the moderation effect of anxious attachment on the association between authentic inner compass and resilience.

Predictor	*R* ^2^	*F*	*B*	SE	95%CI
Sex	0.33	10.45	−0.15	0.08	[−0.31, 0.02]
Age			−0.01	0.01	[−0.03, 0.01]
Educational level			0.02	0.07	[−0.10, 0.15]
Financial satisfaction			0.07*	0.03	[0.01, 0.13]
Authentic inner compass			0.24***	0.04	[0.16, 0.33]
Anxious attachment			−0.11*	0.04	[−0.20, −0.03]
Authentic inner compass × Anxious attachment			0.15**	0.05	[0.05, 0.24]

*Note:* *p < 0.05, **p < 0.01, ***p < 0.001.

**FIGURE 3:**
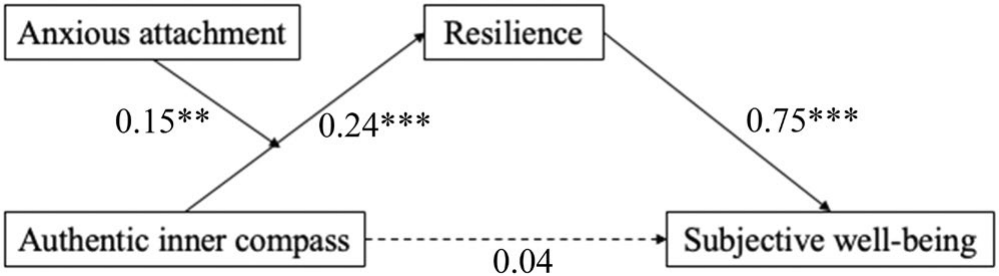
The Moderated Mediation Model. *Note*. Controlling for sex, age, educational level, and financial satisfaction. *p < 0.05, **p < 0.01, ***p < 0.001.

Moreover, as shown in Table 4, the indirect effect of AIC on SWB through resilience was not significant for those with low levels of anxious attachment (*B =* 0.08, SE = 0.07, 95% CI = [−0.02, 0.24]). However, it was significant for those with medium (*B =* 0.18, SE = 0.06, 95% CI = [0.08, 0.32]) or high levels of anxious attachment (*B* = 0.28, SE = 0.08, 95% CI = [0.13, 0.46]). The main results were illustrated in Figure 3.

**TABLE 4:** Conditional indirect effects of authentic inner compass on subjective well‐being via resilience at different levels of anxious attachment.

Level of anxious attachment	Indirect effect via resilience		
	*B*	SE	95% CI
Low (mean − 1SD)	0.08	0.07	[−0.02, 0.24]
Medium (mean)	0.18	0.06	[0.08, 0.32]
High (mean + 1SD)	0.28	0.08	[0.13, 0.46]

## 4 Discussion

Building on the self‐determination theory (Ryan and Deci 2017), this research studied the association between AIC and SWB and the underlying mechanisms among Chinese emerging adults. The results showed that AIC was positively associated with SWB, and resilience fully mediated the association. Importantly, anxious attachment moderated the relationship between AIC and resilience such that this relationship was more salient in more anxiously attached individuals. The findings enrich the self‐determination theory and yield valuable insight into the practices promoting resilience and SWB.

First, the positive correlation between AIC and SWB among Chinese emerging adults supports our first hypothesis and aligns with previous research demonstrating AIC's role in promoting well‐being (e.g., Assor et al. 2021; Russo‐Netzer and Shoshani 2020). It also corroborates the self‐determination theory's assertion that autonomy is a universal need. It matters for individuals' well‐being in cultures with varying autonomy emphases (Ryan and Deci 2017). While previous AIC research has primarily focused on adolescents in autonomy‐oriented cultures, our findings demonstrate that AIC's benefits extend to emerging adults in collectivist contexts. The result underscores the value of fostering AIC in Chinese emerging adults. To promote AIC development, society and educators need to first recognize its universal significance in fostering personal growth and well‐being. Once this recognition is in place, it becomes possible to integrate AIC‐promoting practices—such as offering choices to enhance autonomy, modeling value‐aligned behaviors, and supporting the exploration of authentic personal values (e.g., Assor et al. 2020a, 2020b)—into collectivist settings in a culturally sensitive way, ultimately supporting both individual and community flourishing. More research is needed to develop culturally tailored interventions for promoting AIC.

Our results also revealed that resilience mediates the relationship between AIC and SWB (Hypothesis 2), aligning with insights from the self‐determination theory and resilience literature. Self‐determination theory posits that autonomy satisfaction strengthens internal resources and enhances well‐being (Vansteenkiste and Ryan 2013). Individuals with AIC experience greater autonomy satisfaction, contributing to resilience‐building blocks, such as positive emotion and self‐esteem (Assor et al. 2021; Cohn et al. 2009; Xu et al. 2021). Resilience, in turn, promotes SWB by empowering individuals to navigate challenges effectively (e.g., Chen 2016; Smith et al. 2013). This study advances existing literature by identifying resilience as a critical pathway through which AIC influences SWB, offering new insights into the mechanisms linking autonomy to well‐being. Practically, these findings highlight the value of fostering AIC to build resilience in emerging adults. Given resilience's significant role in enhancing SWB, incorporating autonomy‐supportive practices to strengthen resilience can be an effective strategy for enhancing overall well‐being.

Furthermore, the results revealed that anxious attachment moderates the relationship between AIC and resilience, with AIC showing a stronger positive effect on resilience among highly anxiously attached individuals (Hypothesis 3). This suggests that AIC, by promoting internal resilience resources such as self‐esteem and adaptive coping (Assor et al. 2020a, 2021), may serve as a compensatory mechanism for anxiously attached individuals who often experience relationship dissatisfaction (Mikulincer and Shaver 2019) that undermines their access to external resilience resources (Fergus and Zimmerman 2005; Masten 2018). In contrast, the minimal impact of AIC on resilience among individuals with low anxious attachment suggests a potential ceiling effect, as these individuals likely possess well‐developed external resilience resources due to fewer interpersonal difficulties, which makes them less reliant on AIC to develop resilience. This finding enhances our understanding of how internal and external resilience resources interact and contributes to self‐determination theory by highlighting that autonomy's benefits may be particularly important for individuals facing interpersonal challenges. Practically, these results underscore the value of fostering AIC in individuals with anxious attachment to enhance resilience and improve SWB. In clinical settings, interventions that support the development of AIC could be especially effective in helping anxiously attached individuals build adaptive capacities.

However, the moderation effect of anxious attachment in the AIC‐resilience relationship may vary across cultural contexts. In collectivist cultures like China, where interpersonal relationships are highly valued (Hofstede 2011), the impact of interpersonal relationships on resilience may be stronger compared to individualistic cultures. In these contexts, attachment style may play a more significant role in resilience, as it directly affects relationship dynamics. In contrast, in individualistic cultures, where independence and self‐reliance are emphasized (Hofstede 2011), personal internal resources may be more crucial to resilience, potentially reducing the influence of attachment style. Therefore, the interaction between AIC and attachment styles is likely to differ based on cultural context.

The present study has some limitations. First, employing a cross‐sectional design, this study cannot determine the direction of the studied effects. Future studies could conduct longitudinal designs to determine the temporal relationships among the studied variables or adopt the experimental design to establish causality. Second, the study adopted online self‐report questionnaires, which could be affected by response bias and environmental interference. Additionally, the sample was restricted to Chinese emerging adults, limiting the generalizability of the findings to other cultural contexts or age groups. Future research could examine whether the current findings hold across different cultural contexts and among different populations, especially the moderating effect of anxious attachment.

In summary, we found that AIC was positively associated with SWB via resilience among Chinese emerging adults, and the association between AIC and resilience was more salient for anxiously attached individuals. This study sheds light on the pivotal role of AIC and resilience in promoting SWB and particularly emphasizes the significance of AIC for anxiously attached individuals. By revealing both the mediating mechanisms and boundary conditions within a collectivist cultural context, this study broadens our understanding of how autonomy promotes well‐being across cultures, supporting the cross‐cultural applicability of self‐determination theory. Future practices designed to enhance SWB among Chinese emerging adults could promote AIC and resilience and take individual attachment styles into account.

## Ethics Statement

This study involves human participants and was reviewed and approved by the City University of Hong Kong [SSB5790‐202203‐09]. Participants' informed consent was obtained through an online form.

## Conflicts of Interest

The authors declare no conflicts of interest.

## Data Availability

The data that support the findings of this study are available from the corresponding author upon request.
